# Hypoallergenicity of a hydrolyzed rice protein-based formula containing 2-fucosyllactose and lacto-N-neotetraose in children with cow’s milk allergy: protocol for a randomized controlled study. The RIGHT-HY study

**DOI:** 10.3389/fnut.2026.1798624

**Published:** 2026-04-22

**Authors:** Boutaina Zemrani, Nicholas P. Hays, Enza D’Auria, Francesca Mori, Birgit Kalb, Noura Darwish, Susanna Esposito, Agnieszka Brzozowska, Urszula Jedynak-Wasowicz, Kirsten Beyer, Hania Szajewska, Roberto Berni Canani

**Affiliations:** 1 Scientific Translation and Clinical Strategy Unit, Société des Produits Nestlé S.A., Vevey, Switzerland; 2 Department of Biomedical and Clinical Sciences, University of Milan, Milan, Italy; 3 Allergy Unit, Buzzi Children’s Hospital, Milan, Italy; 4 Allergy Unit, Meyer Children’s Hospital IRCCS, Florence, Italy; 5 Department of Pediatric Respiratory Medicine, Immunology and Critical Care Medicine, Charité – Universitätsmedizin Berlin, Berlin, Germany; 6 Biostatistics, Clinical Research Unit, Nestlé Research, Société des Produits Nestlé S.A., Lausanne, Switzerland; 7 Pediatric Clinic, Department of Medicine and Surgery, University Hospital of Parma, Parma, Italy; 8 Department of Pediatrics and Allergy, Copernicus Memorial Hospital, Medical University of Lodz, Lodz, Poland; 9 Department of Pediatrics, Children’s University Hospital, Jagiellonian University Medical College, Krakow, Poland; 10 German Center for Child and Adolescent Health (DZKJ), Partner Site Charité Universität Berlin, Berlin, Germany; 11 Department of Pediatrics, The Medical University of Warsaw, Warsaw, Poland; 12 Pediatric Allergy Program, Department of Translational Medical Science, University of Naples “Federico II”, Naples, Italy; 13 ImmunoNutrition Lab, CEINGE Advanced Biotechnologies, University of Naples “Federico II”, Naples, Italy; 14 NutriTechLab, University of Naples “Federico II”, Naples, Italy; 15 European Laboratory for the Investigation of Food-Induced Diseases, University of Naples “Federico II”, Naples, Italy; 16 Task Force for Microbiome Studies, University of Naples “Federico II”, Naples, Italy

**Keywords:** children, cow’s milk allergy, human milk oligosaccharides, plant-based formula, rice proteins

## Abstract

Hydrolyzed rice formulas (HRF) are a suitable option for the nutritional management of non-breastfed children with cow’s milk allergy (CMA). Human milk oligosaccharides (HMOs) enhance gastrointestinal health, promote the growth of beneficial gut microbiota, and may reduce the incidence of infections and antibiotic use. There is currently no evidence regarding the hypoallergenicity of HRF containing HMOs. The purpose of this study is to demonstrate the hypoallergenicity of a new hydrolyzed rice protein-based formula containing two manufactured HMOs (HRF-HMO). RIGHT-HY is a multi-center randomized controlled study enrolling infants and children (60 days–3 years) with documented Immunoglobulin E-mediated CMA. Children will be randomized in a crossover fashion to double-blind placebo-controlled food challenges (DBPCFC) with HRF-HMO and an amino acid-based formula (AAF)-HMO, followed by a 7-days open challenge with HRF-HMO requiring a daily minimum intake of 240 ml. Both formulas contain 2-fucosyllactose and lacto-N-neotetraose (1.5 g/L). The primary objective is to demonstrate with 95% confidence that 90% of children with CMA do not develop allergic reactions to HRF-HMO under DBPCFC conditions. Up to 67 children will be enrolled to meet the hypoallergenicity criteria. This sample size allows for a 10% drop-out rate and occurrence of one allergic reaction. An interim analysis will be performed after 42 children have completed both food challenges to assess whether hypoallergenicity criteria have been met, in which case, the study will be stopped for success. Secondary endpoints include HRF-HMO intake and gastrointestinal tolerance during the week-long open challenge, and adverse events throughout the study. The RIGHT-HY study was approved by ethical committees of all participating sites. This study will be the first to generate robust evidence on the hypoallergenicity of this rice formula with added HMOs in children with CMA. The results of the study will be disseminated in peer-reviewed publications and presentations at scientific conferences.

**Study protocol registration:**

https://clinicaltrials.gov/study/NCT06633289, identifier NCT06633289.

## 1 Introduction

Cow’s milk allergy (CMA) is one of the most frequently encountered food allergies in children, affecting an estimated 0.5% to 7.5% of the global pediatric population (1–3). Breastmilk is the ideal source of nutrition for all children including for those with CMA (1). Non-breastfed children diagnosed with CMA should receive a hypoallergenic and nutritionally complete formula. Inappropriate management of CMA can lead to compromised growth and development in these children (4, 5).

Extensively hydrolyzed formulas (eHF) and amino acid-based formulas (AAF) have historically been utilized for non-breastfed infants diagnosed with CMA. Milk from sheep, goat, or buffalo is not suitable for infants with CMA due to high cross-reactivity between the proteins found in those mammalian milks and in cow’s milk (6). Soy-based formulas were the first plant-based formula used for CMA but are generally not recommended in infants below 6 months of age due to concerns regarding phytoestrogen content and risk of co-allergy (6).

Since the 2000s, hydrolyzed rice formula (HRF) were developed, and are now recommended by most scientific societies as a suitable plant-based alternative to cow’s milk-derived eHF for the dietary management of immunoglobulin E (IgE) and non-IgE-mediated CMA when breastfeeding is not possible (7). The European Society of Pediatric Gastroenterology, Hepatology and Nutrition (ESPGHAN) (1) and the World Allergy Organization Diagnosis and Rationale for Action against Cow’s Milk Allergy (DRACMA) (2) recommend the use of HRF for the management of non-breastfed infants with CMA, while the Global Allergy and Asthma European Network (GA^2^LEN) Task Force (8) makes no recommendation for or against hydrolyzed plant-based formulas for managing food allergy in infancy. There has been an increased demand for plant-based alternatives in general, including for the pediatric patients affected by CMA. Rice proteins have low allergenicity and do not have cross-reactivity with cow’s milk proteins (6, 9, 10). Rice is also an important source of protein providing several essential amino acids (6). HRF are generally fortified with selected amino acids such as lysine and threonine to meet the amino acid needs in the pediatric age (6, 11). The protein derived from rice in these formulas is hydrolyzed to enhance its water solubility (6). Concerns have been raised regarding the arsenic level in rice; however, the arsenic levels in HRF are rigorously controlled and maintained within the safe limits established by the European commission (Regulation EU 2023/915 of 25 April 2023) (12, 13).

The American Academy of Pediatrics (AAP) considers a formula to be “hypoallergenic” if at least 90% of infants with documented CMA tolerate it under double-blind, placebo-controlled conditions (14). These hypoallergenicity criteria are also endorsed by other allergy and nutrition societies (1, 15). Evidence supporting the hypoallergenicity of HRF based on double-blind placebo-controlled oral food challenge is scarce to our knowledge with only two clinical studies identified (16, 17). No studies have evaluated the hypoallergenicity of HRF that include human milk oligosaccharides (HMOs). From a safety and tolerability perspective, HRF without HMOs have been shown to be safe, well-tolerated, and can adequately support growth in healthy children (11, 18) and in those with CMA (19–25). Only one recent single-arm study has investigated the tolerance of a HRF containing one manufactured HMO [2-fucosyllactose (2’FL)] in 27 infants with feeding intolerance symptoms or suspected CMA and showed a good safety profile and adequate growth (26).

Human milk contains HMOs as its third largest solid component, whereas cow’s milk has a minimal presence of these compounds (27). HMOs, a group of diverse oligosaccharides, play a crucial role in supporting gut microbiota, gut health and immune health (28). Among the most prevalent HMOs are 2’FL and lacto-N-neotetraose (LNnT) (29). The incorporation of manufactured 2’FL and LNnT into standard cow’s milk-based formulas was found to positively influence gut microbiota composition, aligning it more closely with that observed in breastfed infants (30). In non-breastfed infants with CMA, HMOs supplementation led to the enrichment of gut bifidobacteria and delayed the shift of the microbiome composition toward an adult-like pattern (31). Importantly, the addition of these HMOs was found to be safe and associated with a lower incidence of infections and a reduced risk of antibiotic use when compared to formulas devoid of HMOs, both in healthy children and in those with CMA (32, 33). While the inclusion of manufactured HMOs in infant formulas is rising due their potential beneficial effects, their incorporation into HRF remains limited. To date, no studies have examined the hypoallergenicity of HRF with 2’FL and LNnT in pediatric patients with CMA.

The purpose of this study is to assess the hypoallergenicity of a new HRF containing two manufactured HMOs (HRF-HMO), structurally identical to human milk oligosaccharides, in the management of infants and toddlers with CMA, according to established criteria of hypoallergenicity. Secondary objectives aim to evaluate the tolerability, intake and safety of HRF-HMO during a home usage phase.

## 2 Methods and analysis

### 2.1 Study design and setting

The RIGHT-HY study is a randomized, double-blind, crossover study, followed by 7-days open-label home usage phase. It involves participation from 11 centers across three countries: Poland, Italy, and Germany. The study will be carried out by investigators with expertise in pediatric allergy, pediatric gastroenterology and general pediatrics across both academic and private institutions. The study (Protocol amendment version 1.0, dated 30 May 2024) was approved by ethical committees of all participating sites, and all procedures will be conducted according to the Helsinki Declaration.

The study design is based on the approach recommended to diagnose food allergies (34). The double-blind, placebo-controlled, oral food challenge (DBPCFC) is considered the gold standard for the diagnosis of food allergy and is usually followed by a 7-days open challenge in usual conditions to detect delayed reactions (14, 34).

**FIGURE 1:**
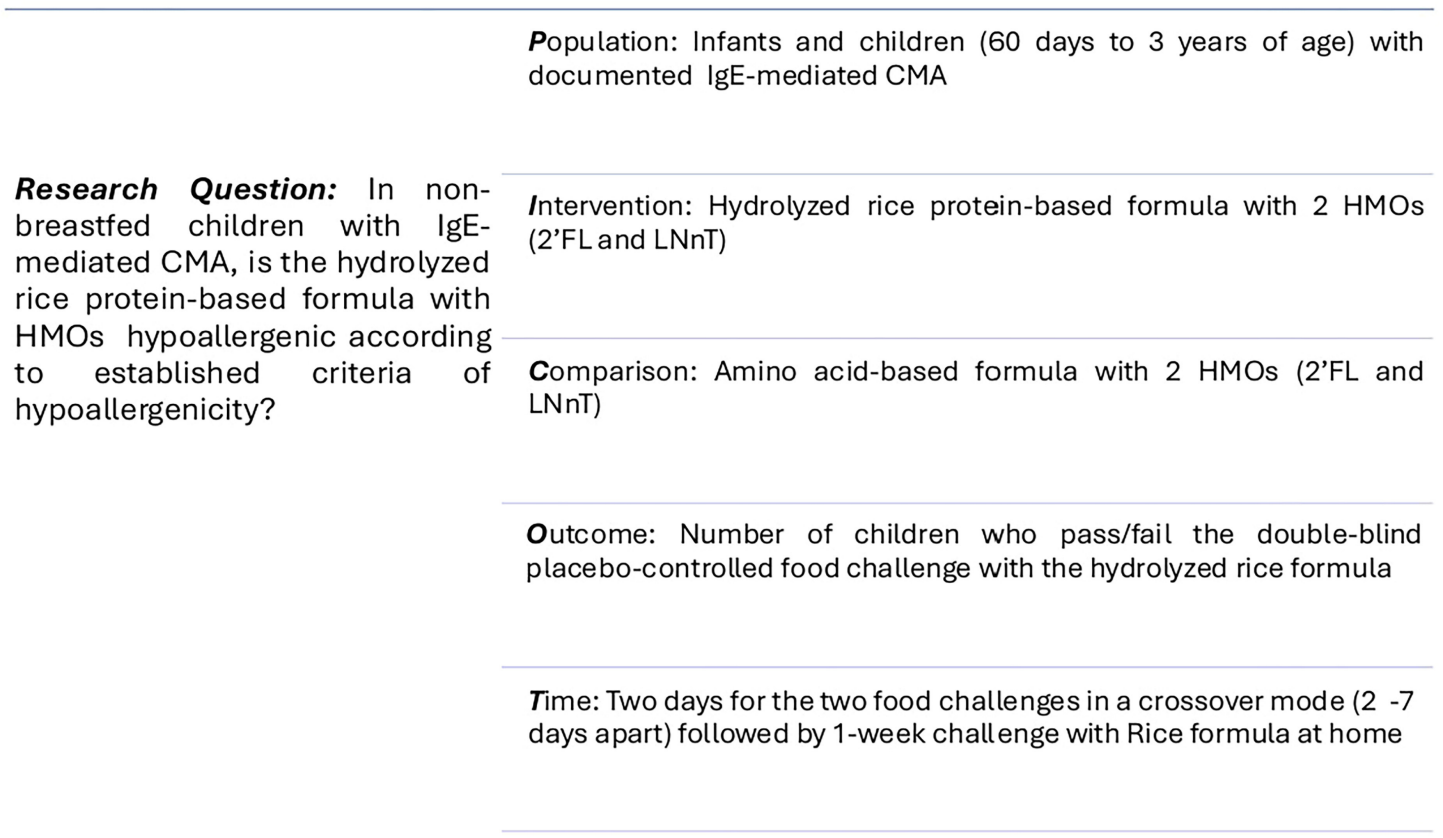
PICOT framework. CMA, cow’s milk allergy; HMO, human milk oligosaccharides; HRF, hydrolyzed rice-protein based formula; IgE, Immunoglobulin E; 2’FL, 2-fucosyllactose; LNnT, lacto-N-neotetraose.

**FIGURE 2:**
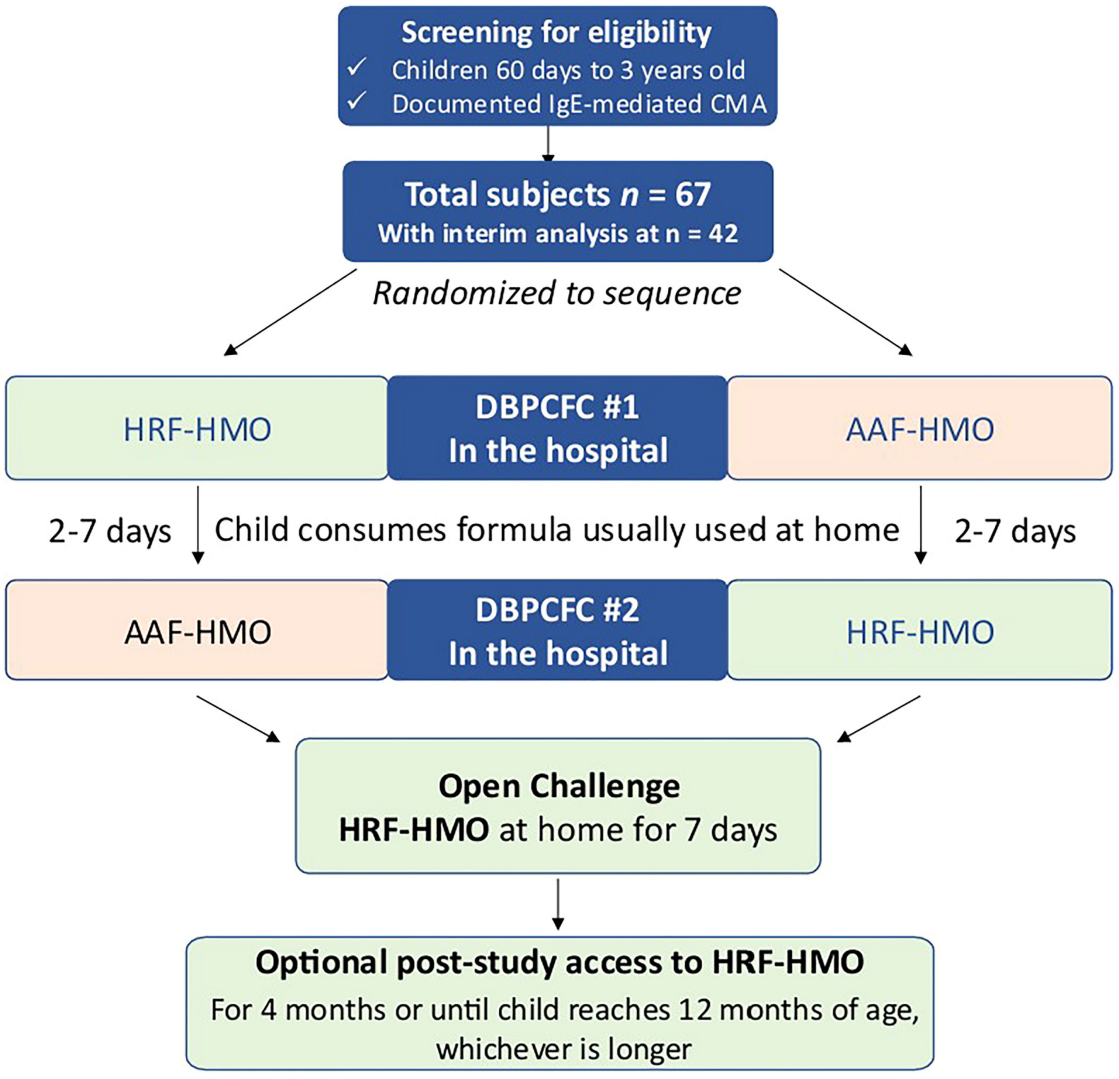
Study flow chart. AAF, amino acid-based formula; CMA, cow’s milk allergy; DBPCFC, double-blind placebo-controlled food challenge; HMO, human milk oligosaccharides; HRF, hydrolyzed rice-protein based formula; IgE, Immunoglobulin E.

The acronym “RIGHT-HY” stands for “Rice-based Infant formula Growth and Hypoallergenicity Trials.” This initiative encompasses two key studies: the current hypoallergenicity study referred to as “RIGHT-HY study” and a growth and safety study, known as the “RIGHT-GO study.” Figures 1, 2 illustrate the PICOT framework and design of the RIGHT-HY study.

### 2.2 Study population

Children who are 60 days–3 years of age with documented IgE-mediated CMA will be eligible for enrollment. IgE-mediated CMA must be documented no more than 6 months prior to enrollment by either (1) presence of convincing immediate allergic symptoms following exposure to cow’s milk confirmed by serum milk-specific IgE (>0.7 kU/L) or positive skin prick test (wheal ≥ 5 mm) or (2) a physician-supervised oral food challenge that elicited immediate allergic symptoms. The 6-months limit aims to reduce the likelihood of enrolling children who may have developed tolerance to cow’s milk over time. Children with documented IgE-mediated CMA between 6 months and 1 year prior to enrollment can be recruited if serum milk-specific IgE > 50 kU/L.

Exclusively breastfed children or those with chronic medical diseases (except atopic eczema), major congenital anomalies or gastrointestinal diseases (other than CMA) will be excluded. Those with known or suspected rice or soy allergy will not be eligible for enrollment. Children with persistent wheezing or chronic respiratory disease, as well as those with severe uncontrolled eczema or treatment by beta-blockers will also be excluded. Strategies for achieving adequate participant enrollment include recruiting patients under the investigators care, and physician referrals.

### 2.3 Sample size considerations

A maximum sample size of 60 children with one allergic reaction to the Test Formula would support the lower bound of the two-sided 95% confidence interval (CI) for the proportion of children who pass the food challenge to be at least 90%, meeting the established hypoallergenicity criteria (14). Allowing for a 10% drop-out rate, approximately 67 children will be enrolled. A sample size of 42 children with no allergic reactions to the HRF-HMO would support the lower bound of the two-sided 95% CI for the non-allergic reaction rate to be at least 90%. Therefore, an interim analysis is planned when 42 completed children in the full analysis set is reached. An external Data Monitoring Committee (DMC), composed of two independent experts in pediatric allergy and one independent expert statistician, will be responsible for reviewing the interim analysis results during a closed session. The DMC will then issue a written recommendation following a pre-specified decision tree stated in the DMC charter. The study may be stopped for success if no reactions have been observed after 42 children complete both food challenges. If one allergic reaction has been observed with the HRF-HMO during the DBPCFC, recruitment will continue to achieve 60 completers of the two food challenges. If two or more allergic reactions are observed with the HRF-HMO during the DBPCFC, the study may be stopped for futility.

### 2.4 Randomization and blinding

Upon obtaining informed consent by the investigators, eligible children will be randomized to one of the two following sequences for the DBPCFC: HRF-HMO followed by AAF-HMO or AAF-HMO followed by HRF-HMO. Randomization will be conducted in a 1:1 ratio using the Medidata Rave “Randomization and Trial Supply Management” (RTSM) technology. RTSM system automates patient randomization, ensures allocation concealment and maintains study blinding.

During the food challenge, the identity of the products will be blinded to the children, caregivers, investigators, site personnel, Contract Research Organization managing the study, and the sponsor (except the manufacturing site, supply, and quality managers). Study formulas will be prepared and administered to participants by a staff member who is not involved in the study assessments and is trained on proper preparation. The products will be packaged and labeled identically but will be distinguishable by unique codes affixed on the primary packaging. Individual coding of cartons will be used to prevent unblinding in case of emergency unblinding. Unblinding of individual coding may be requested by investigators in case of serious adverse events suspected to be related to the investigational product and requiring knowledge of the study product administered. The home usage phase is an open-label phase without blinding.

### 2.5 Interventions

#### 2.5.1 Intervention arm

The Test formula (HRF-HMO) is a hydrolyzed rice protein-based formula without lactose, containing two manufactured HMOs structurally identical to human milk oligosaccharides, 2’FL and LNnT, at 1.5 g/L in a 2:1 ratio, and medium-chain triglycerides. This level and ratio of HMOs are comparable to those found in human milk. The protein content is 2.3 g/100 kcal with a Protein Digestibility-Corrected Amino Acid Score (PDCAAS) of 1. The arsenic content of the formula is maintained within safe limits, below the maximum inorganic arsenic content for “Food for Special Medical Purposes” (FSMPs) intended for infants and young children under the age of 3 set by the European commission (Regulation (EU) 2023/915 of 25 April 2023) (35). The macronutrient and HMO composition of the study formulas is presented in Table 1.

**TABLE 1:** Macronutrient and HMO composition of study formulas.

Formula composition	Hydrolyzed rice protein-based formula (HRF-HMO)	Amino acid-based formula (AAF-HMO)
Caloric density (kcal/100 ml)	67.5	66.1
Protein (g/100 kcal)	2.3*	2.67
Protein (g/100 mL)	1.55	1.8
Protein source	Hydrolyzed rice protein	Free amino acids
Lipids (g/100 kcal)	5.2	4.9
Carbohydrate (g/100 kcal)	10.8	11.1
Carbohydrate source	Maltodextrin, starch	Maltodextrin, starch
2’FL (g/L)	1.0	1.0
LNnT (g/L)	0.5	0.5

AAF-HMO, amino acid-based formula containing two manufactured HMOs; HRF-HMO, hydrolyzed rice protein-based formula containing two manufactured HMOs; LNnT, lacto-N-neotetraose; 2’FL, 2-fucosyllactose. *Minimum protein value of 2.25 g/100 Kcal.

#### 2.5.2 Control arm

The control formula (AAF-HMO) is a commercially available amino acid-based formula (Alfamino HMO^®^, Nestlé Health Science, Vevey, Switzerland) without lactose, containing medium-chain triglycerides and the same amount and type of HMOs as the Test HRF-HMO. The protein content of the control AAF-HMO is 2.67 g/100 kcal. This formula was selected as a control as it is completely devoid of cow’s milk proteins and has been shown to be hypoallergenic (36).

The Test and Control formulas are both considered FSMPs intended for infants with CMA. Both formulas are nutritionally complete and fall in the scope of Delegated Regulation (EU) 2016/128. The formulas will be provided in powder form and administered orally after reconstitution with water, using an infant feeding bottle, cup, bowl, or other container depending on age and developmental stage.

### 2.6 Study procedures

Study visits (V) and procedures are shown in Table 2. After enrollment, there will be 2 visits corresponding to the two oral food challenges, scheduled 2–7 days apart, followed by a final visit 1–2 weeks after the end of the at-home phase.

**TABLE 2:** Study visits and procedures.

Visit	V1	V2	V3	Open challenge	V4
Study day	Study day 0	V1 + 3–28 days	V2 + 2–7 days	After V3 for 7 days	V3 + 8–14 days
Informed consent	X				
Assign subject number	X				
Inclusion-exclusion criteria	X				
Demographic information	X				
Medical history	X				
Physical exam, weight, vital signs		X	X		X
Height/length	X				
Randomization		X			
DBPCFC		X	X		
Product dispensing			X		X (if desired)
HRF-HMO administration				Daily	Check compliance
Digestive symptoms and formula intake record				Daily	Check record
Cow’s milk elimination diet	→	→	→	→	→
Concomitant diet and treatment	→	→	→	→	→
Adverse event record	→	→	→	→	→

DBPCFC, double-blind placebo-controlled food challenge; HRF-HMO, hydrolyzed rice protein-based formula containing two manufactured HMOs; V, visit.

#### 2.6.1 Double-blind placebo-controlled food challenge (DBPCFC)

The food challenge will be performed in a healthy child and will be postponed if the child is sick or if antihistamines or oral steroids were used 7 and 14 days before the challenge, respectively. The timing of the DBPCFC will be planned around the child’s normal schedule. Parents will be advised to feed their child a light non-fatty meal (about half of the usual portion) approximately 2 h before each test, and no foods or drinks should be consumed after this meal until the completion of the test procedure. Parents will also maintain a strict cow’s milk elimination diet for their children throughout the study period including during the DBPCFC.

During the food challenge, children will be fed increasing volumes of formula at intervals of at least 20 min, until reaching the target volume. The total volume is equivalent to an age-appropriate serving size as per expert recommendations (34, 37, 38), and will be a minimum of 180 mL (up to 210 mL) for infants below 1 year, and a minimum of 200 mL (up to 240 mL) for children ≥1 year, according to their usual serving size. If a child is not able to drink the total volume, a re-attempted feeding after a short break or a repeat of the test on a later date will be proposed, at the discretion of the investigator. Table 3 summarizes the dosing schedule during the food challenge, per age group.

**TABLE 3:** Formula volumes and corresponding protein doses per age group.

Dose number	Infants < 1 year		Children ≥ 1 year		Timepoint (minutes)
	Protein dose (mg)	Feeding volume (mL)	Protein dose (mg)	Feeding volume (mL)	
1	31	2	31	2	0
2	101	7	101	7	20
3	307	20	307	20	40
4	920	60	1074	70	60
5	1381–1841	90–120	1534–2148	100–140	80
Cumulative dose/volume	2740–3200 mg	∼180–210 mL	3047–3661 mg	∼200–240 mL	1 h 20 min

Before each dose, vital signs will be measured and a physical examination completed to determine whether any change in the child’s clinical status has occurred in conjunction with the DBPCFC.

The following definitions will be used for to determine the outcome of the DBPCFC:

- To pass the food challenge means to consume the total volume of formula and display no dose-limiting allergic reaction to any of the doses.
- To fail the food challenge means to display a dose-limiting allergic reaction during the food challenge.
- To complete the food challenge means to either pass or fail the test.

**FIGURE 3:**
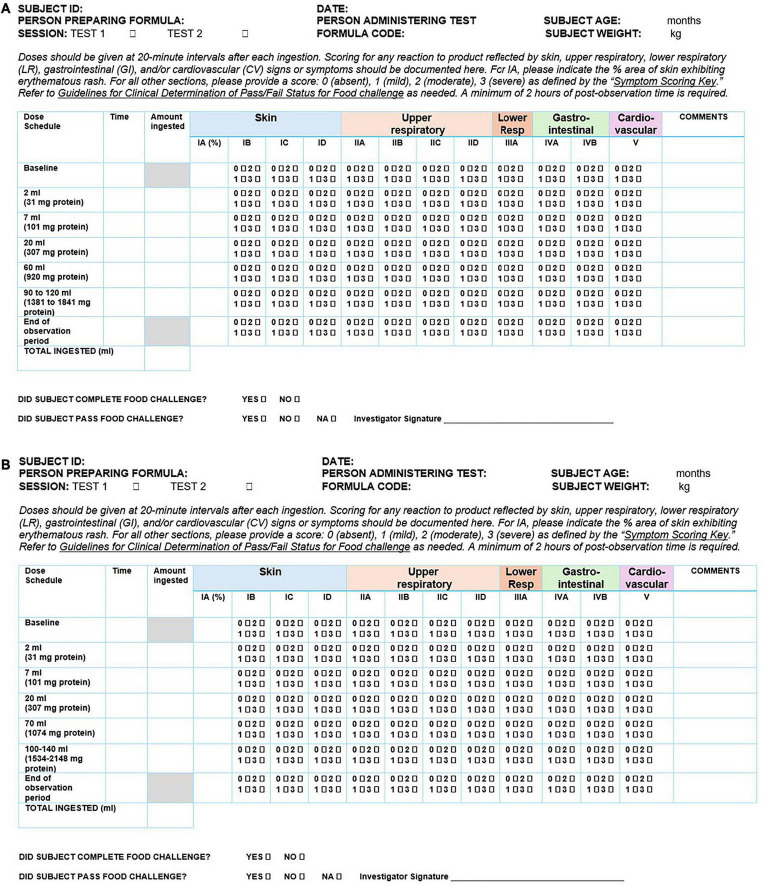
Data collection forms for the oral food challenge. **(A)** Data collection form for the oral food challenge for infants below 1 year of age. **(B)** Data collection form for the oral food challenge for children above 1 year of age.

Investigators will document any allergic symptoms (skin, gastrointestinal, respiratory, cardiovascular) occurring during the DBPCFC on a standardized data collection form and will adhere to a common definition of the criteria for the pass/fail for each symptom during the DBPCFC (38). A minimum post-test observation period of 2 h will be required. Figure 3 and Table 4 show the instructions for the clinical determination of the pass/fail status for the oral food challenge and the data collection form, respectively.

**TABLE 4:** Instructions for clinical determination of pass/fail status for the food challenge.

	Symptoms	0 None	1 Mild	2 Moderate	3 Severe
Skin	IA skin rash, erythematous rash –% area involved				
	IB pruritis	Absent	Occasional scratching	Scratching continuously for > 2 min at a time	Hard continuous scratching–excoriation
	IC urticaria/angioedema	Absent	<3 hives	3 –10 hives Lip edema	Generalized involvement Significant angioedema
	ID rash	Absent	Few areas of faint erythema	Areas of erythema, macular and raised rash	Marked erythema (>50%), extensive raised lesion (>25%), vesiculation and/or piloerection
Upper respiratory	IIA sneezing	Absent	Rare bursts	Bursts < 10, intermittent rubbing of nose, and/or eyes	Continuous rubbing of nose and/or eyes, periocular swelling and/or long bursts of sneezing
	IIB nasal congestion	Absent	Some hinderance to breathing	Nostrils feel blocked/breathes through mouth most of time	Nostrils occluded
	IIC rhinorrhea	Absent	Occasional sniffling	Frequent sniffling, requires tissues	Nose runs freely despite sniffling and tissues
	IID laryngeal	Absent	Throat clearing, occasional cough	Hoarseness, frequent dry cough	Inspiratory stridor
Lower respiratory	III wheezing	Absent	Expiratory wheezing to auscultation	Dyspnea, inspiratory and expiratory wheezing	Dyspnea, use of accessory muscles, audible wheezing
Gastro-intestinal	IVA subjective	Absent	Complaints of nausea or abdominal pain, no change in activity, itchy mouth	Frequent c/o nausea or pain, decreased activity	Subject in bed, crying or notably distressed
	IVB objective	Absent	1 episode of diarrhea (ensure no diarrhea prior to challenge) or emesis	2–3 episodes of emesis or diarrhea or 1 of each	>3 episodes of emesis or diarrhea or 2 of each
Cardio-vascular	V cardiovascular	Normal heart rate, or BP for age/baseline	Color change, subjective response (weak, dizzy), mental status change, sustained tachycardia	Drop in blood pressure and/or >20% drop from baseline	Cardiovascular collapse, signs of impaired circulation (unconscious)
Pass/Fail Status:					
**GREEN**		Continue; Pass			
**YELLOW**		Pass/fail status based on clinical judgment. If symptoms occur mid-test, consider longer observation before next dose, and/or repeat the dose rather than increasing the dose. If no progression of symptoms, could continue with further dosing. If no additional symptoms develop, this could be considered a passed test.			
**RED**		STOP: failed test if: 1. Mild lower respiratory, laryngeal, or objective gastrointestinal symptoms 2. Any moderate or severe symptoms 3. Two or more mild symptoms from different organ systems 4. Any challenge that resulted in treatment of allergic symptoms			

#### 2.6.2 Home usage phase

All children who pass both DBPCFC will receive the HRF-HMO during 7 days at home, with instructions to drink a minimum of 240 ml daily. This home phase will support the hypoallergenicity and document gastrointestinal tolerance of this new formula. During this period, caregivers will record daily formula intake in a diary, as well as gastrointestinal symptoms and adverse events. After the at-home open challenge, caregivers will be offered post-study access to HRF-HMO for 4 months use or until the child is 12 months of age. This continued access to the study formula is intended to ensure the health and well-being of study participants who may still need access to a hypoallergenic formula that has been identified as beneficial for that participant during the study.

### 2.7 Outcomes

#### 2.7.1 Primary outcome measure

The primary outcome is the number of children who pass the DBPCFC with HRF-HMO. Passing a food challenge means the total volume of formula is consumed and no dose-limiting allergic reaction to any of the doses is displayed. Failing a food challenge means displaying a dose-limiting allergic reaction during the food challenge. If a child does not achieve the total volume consumption because of a reason other than an allergic reaction, such as inability to drink the formula, this will be considered an incomplete challenge, not a failure to the DBPCFC. Standardized criteria will be used by all investigators to assess the “pass/fail status” of the food challenge (34, 37, 38).

The primary outcome is based on international criteria of hypoallergenicity (14). A formula is considered hypoallergenic if at least 90% of children with CMA can tolerate it with no allergic reactions (14).

#### 2.7.2 Secondary outcomes

##### 2.7.2.1 Gastrointestinal tolerance during the home phase

Gastrointestinal tolerance symptoms will be recorded daily during the at-home open challenge. They include frequency and severity of flatulence, vomiting or discomfort, as well as stool frequency, difficulty passing stools, and stool consistency according to the Brussels Infant and Toddler Stool Scale (39).

##### 2.7.2.2 HRF-HMO intake during the home phase

Intake of HRF-HMO will be recorded daily during the home phase. Children will be asked to drink a minimum of 240 ml of HRF-HMO daily, either once or in smaller servings throughout the day, depending on the child’s eating habits and appetite.

##### 2.7.2.3 Adverse events

Adverse events will be collected from the time informed consent is signed until the end of the at-home open challenge. Evaluation of potential adverse events will be performed during each visit by study investigators. The type, severity, seriousness, duration and relationship of the adverse events to study formulas will be recorded and listed by system-organ class and preferred term according to the Medical Dictionary for Regulatory Activities (MedDRA) glossary. During the period where caregivers are offered post-study access to the rice-based formula, adverse events can be spontaneously reported by parents.

### 2.8 Statistical analysis

The analyses of this study will adhere to the recommended guidelines outlined in the CONSORT 2022 statement for reporting outcomes in clinical trial reports (40).

Dataset populations include the intention to treat (ITT) analysis population (all randomized children), the safety analysis (SAF) population (ITT population with documentation of at least one administration of study product), full analysis (FAS) population (all randomized children completing both food challenges), and the per protocol (PP) population (FAS subjects completing the study with no major protocol violations which are believed to impact the primary endpoint).

#### 2.8.1 Primary outcome

The number and percentage of children who pass/fail the DBPCFC with the HRF-HMO will be presented. The two-sided 95% CI, and its corresponding *p*-value will be provided for the point estimates of the proportion of children with no allergic reactions to each formula during the food challenge. At both interim and final analysis, the study will be considered a success if the lower bound of the two-sided 95% confidence interval for the proportion of children who pass the food challenge with HRF-HMO is at least 90%.

#### 2.8.2 Secondary outcomes

Secondary outcomes of gastrointestinal tolerance (stool frequency, stool consistency, gastrointestinal symptoms) collected during the at-home open challenge will be summarized descriptively. The incidence of adverse events and allergy-related symptoms will be summarized by study product (HRF-HMO and AAF-HMO) during the food challenge, and by period for HRF-HMO (during the food challenge and the at-home phase). Occurrence of adverse events, including any gastrointestinal, skin or respiratory symptoms, will be summarized in a table presenting frequency, severity, seriousness of adverse events, as well as their relationship to the assigned formulas.

The results of the primary and secondary outcomes will be presented in the ITT and PP populations.

#### 2.8.3 Data management

Data management systems used in the RIGHT-HY study will comply with Good Clinical Practices, laws, and regulations. Data will be entered from the source document into an electronic Case Report Forms by the investigators. The gastrointestinal symptoms and formula intake record will be filled by the caregivers using paper questionnaires. The Clinical Data Manager will validate all data entered into the eCRF. Data discrepancies will trigger automatic queries, and if necessary, manual queries will be sent to the Investigator for clarification. All data modifications will be documented in an audit trail file.

Data will be periodically and blindly monitored throughout the study by the sponsor representatives to evaluate study progress and to verify quality and completeness of data collection.

## 3 Discussion

We present the first study protocol evaluating the hypoallergenicity of a HRF containing two manufactured HMOs intended for the management of infants and children with CMA. The results of this study will support the safe use of HRF in children with CMA and provide evidence that this new HRF with HMOs meets the generally accepted criteria of hypoallergenicity. Providing families of non-breastfed infants with plant-based options that are not only palatable, but also supported by documented efficacy and safety is important.

The criteria for defining a formula as hypoallergenic have been established (14), and the design of this study adheres to those requirements. The rate of allergic reactions to the HRF-HMO will determine its hypoallergenicity under double-blinded controlled conditions. The 1-week open challenge phase will document the safety and gastrointestinal tolerance of the HRF-HMO in typical home use. Additional data on the growth, safety and tolerability of HRF-HMO will be collected over a 4-months period in a complementary study (NCT06633250), including an assessment of allergy symptoms and quality of life.

The addition of HMOs to HRFs could play a critical role in supporting gut health and immune health of non-breastfed infants with CMA. Preclinical data suggest that HMOs might attenuate allergic symptoms (41) and might promote tolerance development via interaction with dendritic cells (42). In clinical studies, the addition of 2’FL and LNnT to an eHF formula partially corrected the gut dysbiosis commonly seen in infants with CMA, which may affect the maturation of their immune system (31, 43, 44). Furthermore, the manufactured HMOs included in this formula could have a beneficial impact on the reduction of infections and antibiotic use, as observed in studies involving eHF and HMOs. These HMOs may also positively impact the long-term health trajectories of formula-fed infants. As popularity and usage of HRFs grow, future studies should investigate the impact of HMOs within the HRF matrix on other relevant outcomes such as clinical infections and changes in gut microbiota.

The main strengths of this study lie in its rigorous methodology and novelty. Notably, the study features a well-designed food challenge protocol, in line with recommendations for hypoallergenicity studies. The randomized, controlled, double-blind design minimizes the risk for bias and strengthens the validity of study results. Furthermore, the multicenter and multinational approach of the study will enhance the generalizability of the findings. Importantly, this is the first study to generate data on the hypoallergenicity of a HRF containing HMOs. Limitations of this study include the use of an AAF as a control during the DBPCFC, which may affect the ability of some children to consume it during the food challenge due to its specific taste. However, the use of AAF is considered a safer option during the food challenge as up to 10% of children with CMA may experience an allergic reaction to eHFs.
